# The role of perspective‐taking in attenuating self‐group distancing in women managers

**DOI:** 10.1111/bjso.12812

**Published:** 2024-10-28

**Authors:** Janine Bosak, Clara Kulich, Samantha C. Paustian‐Underdahl, Rachelle Borg Dingli

**Affiliations:** ^1^ Dublin City University Dublin Ireland; ^2^ University of Geneva Geneva Switzerland; ^3^ Florida State University Tallahassee Florida USA; ^4^ Independent Researcher Rabat Malta

**Keywords:** perspective‐taking, power, queen bee, self‐ingroup distancing, social identity threat, women leaders

## Abstract

Contrary to expectations about solidarity and sisterhood between women, women managers sometimes distance themselves from junior women in the workplace when facing identity threat, that is, the feeling that one's social identity—such as race or gender—is devalued or undermined. For example, women managers might distance themselves from lower status junior women by seeing themselves as more masculine and career committed than their junior women colleagues. To advance our understanding of how to combat self‐group distancing, the present research proposed and tested whether taking the perspective of junior women would attenuate these ingroup‐distancing tendencies in women managers. Findings from a field study and an experimental study indicated that women managers reported greater self‐distancing from junior women (on masculine trait perceptions) compared to women employees. As predicted, this effect was attenuated for women managers with high levels of perspective‐taking (Study 1) and for women who were experimentally led to take the perspective of junior women (Study 2). For ratings of career commitment and support for affirmative actions, we did not replicate the self‐ingroup distancing effect reported in the literature. Theoretical and practical implications of these findings are discussed.

## INTRODUCTION

*“Empowered Women Empower Women”*‐Unknown.

This quote reflects the hope that women in high‐status, high‐power positions will empower, support, and act as inspirational role models to other women (Ely, 1995; Ragins & Scandura, 1994). Building on this assumption of solidarity and sisterhood between women (see Mavin, 2006), it may appear logical to conclude that a higher number of women in high‐status, high‐power positions in organizations will foster gender equality in management.

Yet, this solution is compromised by the fact that, under certain conditions, women managers might not act supportive towards more junior women. Some women who achieve high‐status, high‐power roles in male‐dominated organizations may “behave in ways that impede rather than help the advancement of other women” (p. 903, Faniko et al., 2016). For instance, some women managers may propagate harmful gender stereotypes against junior women (e.g., by endorsing stereotypes of junior women as less ambitious and less committed than men) and provide less support to junior women than junior men (e.g., Derks et al., 2016; Kulich et al., 2015; Paustian‐Underdahl et al., 2017). This self‐distancing of women managers from their gender ingroup aids and abets the status quo of women's underrepresentation in leadership roles and diminishes career opportunities for other women (see Ellemers et al., 2012).

As such, scholars and practitioners alike have a vested interest in understanding the circumstances that lead to self‐ingroup distancing and *how* to combat it. The present work responds to the call by Derks et al. (2016; p. 465) to “focus on discovering ways to eliminate this dynamic” and adds to the scarce amount of research on strategies for reducing self‐ingroup responses (e.g., Derks et al., 2009; Derks et al., 2011). Across two studies, we propose and test perspective‐taking as a factor that may prevent self‐ingroup distancing. Our findings yield important implications for both theory and practice by enriching our understanding of ingroup distancing tendencies and informing interventions in organizations that seek to prevent or attenuate potentially harmful self‐ingroup distancing, respectively.

### Self‐group distancing

Self‐group distancing among women–originally coined, “the queen bee” syndrome (Staines et al., 1974)–can take different forms. In previous research that focused on women in power and the work context, self‐group distancing of women managers was examined in three ways (e.g., Derks et al., 2016): (a) distancing from junior women, by rating their own career commitment higher than that of junior women; (b) assimilation to the higher‐status group of men, by providing highly masculine self‐presentations; and (c) legitimizing the current status quo of gender imbalance in power, status, and leadership, by objecting to policies that would address gender inequalities and improve women's current status. Scholars argue that such reactions are context‐dependent, a consequence of various challenges experienced by women rising to leadership in organizations (e.g., Paustian‐Underdahl et al., 2017), and due to competition between women brought about by scarce resources and opportunities in the workplace (see Sheppard & Aquino, 2013).

From a social identity perspective (Tajfel & Turner, 1986), self‐group distancing behaviour could be regarded as “an individual mobility response to the identity threat that women may encounter at work” (p. 904, Faniko et al., 2016). Discriminatory or masculine organizational contexts or roles can be threatening environments for women managers if they perceive that their gender identity is devalued. A woman who has risen in the social hierarchy through means of professional mobility has to manage two conflicting identities: her ascribed low‐status identity of being a woman and her achieved high‐status identity of being a manager (Chipeaux et al., 2017; Kulich et al., 2015). Self‐group distancing serves as an individual mobility strategy that allows women to focus on the valued professional identity whilst downplaying the less valued identity associated with being a woman. Role congruity theory (Eagly & Karau, 2002) also considers the perceived incongruity between the communal qualities (e.g., kind, affectionate) of the female gender role and the masculine‐agentic (e.g., assertive, independent) qualities of the management role. In contrast to the management role, the lower‐status role of an employee (or follower) has been found to be congruent with the communal qualities of the feminine gender role (Braun et al., 2017; Eagly & Karau, 2002). Hence, women who make it into the management ranks of an organization might experience their feminine gender identity to be at odds with their achieved identity of being a manager.

It follows that continued association with the devalued group can bear a threat to these individuals' identities who try to establish themselves in these higher spheres. One strategy to reduce this threat is for these individuals to define their identity more in terms of traits associated with their new high‐status ingroup (manager) rather than their low‐status group of origin (women) (i.e., to distance themselves from their low‐status ingroup, that is, women in more junior roles; Ellemers, 2001). In short, self‐group distancing reactions are “examples of self‐protecting social mobility responses to identity threat experienced by lower‐status groups in contexts of inequity” see (p. 439, Paustian‐Underdahl et al., 2017).

Previous studies found that women managers showed a tendency to describe themselves as more masculine than junior women in their organizations, to see themselves as more committed to their careers than junior women to theirs, and to support affirmative action less than junior women (see Derks et al., 2016 for an overview). Such self‐group distancing is more likely to exist in male‐dominated settings and in organizations with adverse diversity climates (Derks et al., 2016; Paustian‐Underdahl et al., 2017). Indeed, women managers who experienced or were reminded of gender discrimination showed greater self‐group distancing from other women (Derks et al., 2016), and women employees perceived lower perceived supervisor support from managers of the same gender or racial low‐status ingroup, particularly in adverse diversity climates (Paustian‐Underdahl et al., 2017).

Given the drastic consequences of self‐group distancing for junior women, women leaders, the organization, and the gender hierarchy (for an overview, see Derks et al., 2016), it is imperative to identify and test potential ways for reducing self‐group distancing in the workplace. Up to this point, strategies proposed and examined to combat self‐group distancing have focused on self‐affirmation strategies and reducing the belief in system legitimacy. Self‐affirmation strategies aim to reduce social identity threat, for instance, by providing women with positive feedback from supervisors (e.g., Derks et al., 2011), with this focus on personal values and accomplishments affirming the self‐concept (e.g., Aronson et al., 1999) and thus reducing threat and the need to distance from more junior women. Another set of strategies aims to reduce the belief in system legitimacy, for example, by increasing awareness of personal discrimination which has been found to elicit higher identification with one's group (Branscombe et al., 1999) and is likely to promote collective rather than individual coping strategies. Our investigation focuses on perspective‐taking as a third potential strategy for preventing, or at least reducing self‐group distancing by women managers.

### Reducing self‐group distancing through perspective‐taking

The ability to imagine the world from another's point of view is a fundamental aspect of social functioning (Davis, 1983). It is therefore not surprising that perspective‐taking is associated with social competence (Davis, 1983), moral development (Kohlberg, 1976), empathy and altruism (Cialdini et al., 1997), and decreases in system‐justifying beliefs (Li & Edwards, 2021). Further, perspective‐taking can reduce stereotyping and negative evaluations of outgroup members (e.g., Galinsky & Moskowitz, 2000; Todd et al., 2011). Additionally, studies have demonstrated that the positive effects of perspective‐taking on intergroup evaluations are quite robust and that these effects are evident in the immediate experimental context and persistent over time (e.g., Clore & Jeffery, 1972; Devine et al., 2012).

We believe that perspective‐taking may be especially important for reducing self‐group distancing among women in leadership roles given that power can often lead to outcomes that conflict with perspective‐taking (Galinsky et al., 2006). For example, power was found to reduce compassion (van Kleef et al., 2008), attention to others' emotions (Galinsky et al., 2006), empathic accuracy (Kraus et al., 2010), an accurate understanding of alliances (Brion & Anderson, 2013), and to be associated with social distance (Magee & Smith, 2013). Moreover, women imagining the self in a powerful position reported reduced gender identification and increased endorsement of sexism (Vial & Napier, 2017). Why is it that the powerful attend less to the perspectives of others?

First, individuals in power hold control over valuable resources, and therefore, they are less reliant on others to accomplish goals (Galinsky et al., 2006). Second, power usually comes with increased attentional demands, which makes it more challenging for power holders to adopt the perspective of those under their charge (Fiske, 1993). Finally, whereas perspective‐taking results in others becoming more “self‐like” (Davis et al., 1996), having power fosters psychological distance from other people (Lee & Tiedens, 2001). For women, in addition to these effects, the enactment of power in leader roles mostly happens in identity‐threatening environments (Derks et al., 2016), thus, they may be particularly vulnerable creating even stronger distance from others. These theoretical perspectives and related findings suggest that contrary to ideas about solidarity and sisterhood between women (see Mavin, 2006), women managers who are in positions of power might not act as change agents for junior women in work settings. Yet, perspective‐taking may serve to mitigate this phenomenon, given that power and perspective‐taking can produce synergistic effects, with perspective‐taking steering power holders’ agentic tendencies away from the self to others and with power “propelling people to act on their understanding of others” (p. 628; Galinsky et al., 2014).

To conclude, if we consider that women managers and junior women in work settings form two sub‐categories of their gender group (Faniko et al., 2017), we can assume that women managers (i.e., those with more power), in organizations where most leadership positions are held by men, view junior women as an out‐group and as fulfilling the stereotypical perception of women as being ‘less adequate’ for leadership (Eagly & Karau, 2002). This might contribute to self‐group distancing and detachment of women managers from other, more junior women. We propose here that perspective‐taking may be an effective strategy to reduce self‐group distancing by women managers such that ingroup distancing (in terms of masculine trait ascription, career commitment, and affirmative action support) may be attenuated when they take the perspective of junior women.

## THE PRESENT RESEARCH

The aim of the present research is to investigate (a) the role of hierarchical position on self‐group distancing and (b) the role of perspective‐taking in mitigating these self‐group distancing responses towards junior women. We examined these research questions in two studies‐one field study with public sector employees from Malta (Study 1) and one experimental study with MTurk workers from the United States (Study 2). In our work, the term ‘junior women’ refers to the evaluated target group of women in junior positions in organizations, and the terms ‘employees’ and ‘managers’ refer to the group of women participants who occupy an employee position or a managerial position, respectively.

Building on Faniko et al. (2017), Study 1 tests the role of women's hierarchical position (manager vs. employee) on three self‐group distancing outcomes. In a novel extension of this research, Study 1 also examines the moderating role of perspective‐taking in predicting how women in managerial versus employee positions differ in responses associated with the self‐group distancing phenomenon. Using an experimental design, Study 2 then manipulates perspective‐taking in both women managers and women employees to test whether taking the perspective of ‘junior women’ reduces self‐group distancing of managers towards the target group of junior women. Specifically, we experimentally created a psychological state that is junior women‐focused (vs. self‐focused vs. other‐focused) to establish causally that perspective‐taking through the focus on junior women can attenuate the self‐group distancing responses of managers towards junior women. Overall, we test two specific hypotheses in the present research:

Hypothesis 1 Women managers (compared to women employees) will report stronger self‐distancing from women occupying junior positions. Specifically, managers will rate themselves as more masculine (H1a) and more career committed (H1b) than they will rate junior women; managers will also rate themselves as more masculine and career committed compared to self‐ratings of employees. Moreover, women managers will be less likely to support affirmative actions for (junior) women, compared to women employees (H1c).

Hypothesis 2 Taking the perspective of junior women (vs not) will attenuate the self vs. junior women distancing effect. Specifically, women managers will show less self‐distancing from junior women when taking the perspective of junior women, that is, lower distancing in masculinity (H2a) and career commitment ratings (H2b) and an increase in the support of affirmative action for junior women (H2c).

### Transparency

The de‐identified dataset and analysis code of Study 1 are available by emailing the corresponding author for Study 1. All data, analysis code, and research materials are available on OSF for Study 2 (https://osf.io/vc9ed). Study 2's design, hypotheses, and analysis were pre‐registered (https://osf.io/ajknu) and additional pre‐registered exploratory questions were formulated for the measures included in the Data S1. Ethical approval was obtained for Studies 1 and 2.

## STUDY 1

### Participants

We sent an online survey to 5000 public employees in Malta and obtained 547 completed surveys across all employees (reflective of a response rate of 11%). Respondents were 314 women, 233 men, and 5 individuals who did not indicate their sex. Given the focus of our study on women managers and women employees only, we analysed the data from the 314 women participants (mean age = 40.75, *SD* = 9.66). On average, participants had been working in their current organization for 13.96 years (*SD* = 10.76), with 92.7% working full‐time, 170 (54.1%) women held an employee/non‐managerial position, and 144 (45.9%) women a managerial position.

A priori power analyses using G*power3 (Faul et al., 2007) indicated that achieving 95% power to detect a medium effect size (*f* = 0.25) at α = .05 would require 210 participants in a one‐way analysis of variance (ANOVA) and 119 participants in a regression model with three predictors (*f* ^2^ = 0.15). Sufficient data were subsequently collected.

### Measures

All items were measured using 7‐point Likert rating scales ranging from 1 (strongly disagree) to 7 (strongly agree). Measures are indicated in chronological order. A list of additional exploratory measures and a correlation table between all measures (SM Table S1) can be found in the Data S1.

#### Self‐group distancing indicators

As per previous studies (e.g., Faniko et al., 2017), we measured three indicators of the self‐group distancing phenomenon expressed by women managers towards ‘female junior colleagues’.

##### Perceived masculinity

Four attributes (assertive, having leadership abilities, willing to take risks, dominant) taken from Bem's Sex Role Inventory (Bem, 1974, see also Faniko et al., 2016) measured perceived masculinity. Participants indicated the degree to which these attributes were characteristic of the self (*α* = .71, *M* = 5.09, *SD* = 0.96), and of junior women (*α* = .76, *M* = 4.50, *SD* = 1.09).

##### Perceived career commitment

On three items from Ellemers et al. (1998), see also Faniko et al. (2016), participants rated their personal career commitment (*α* = .90, *M* = 4.36, *SD* = 1.62, e.g., ‘My career is one of the most important things in my life’) as well as the career commitment of junior women at the beginning of their career (*α* = .96, *M* = 4.78, *SD* = 1.45, e.g., ‘The career is one of the most important things in their life’).

##### Support for affirmative action

Participants rated their agreement with seven affirmative action items from Tougas and Veilleux (1988); (*α* = .90, *M* = 5.79, *SD* = 0.97, e.g., ‘I am in favor of implementing training programs designed to give women access to non‐traditional jobs’; ‘I am in favor of implementing an equality of access program’).

#### Perspective‐taking

Perspective‐taking is the tendency to adopt the psychological point of view of others and is often assessed using the interpersonal reactivity index (IRI, Davis, 1980; see for example, Wang et al., 2014). Participants rated their agreement with seven items (*α* = .73, *M* = 5.29, *SD* = 0.74, e.g., ‘I sometimes find it difficult to see things from the other person's’ point of view’, ‘I try to look at everybody's side of a disagreement before I make a decision’).

#### Hierarchical position

Participants reported the organizational level they had achieved among five response options. The option ‘Employee/non‐manager’ (54.1%) became the ‘employee’ category, the four remaining options ‘Team leader/supervisor’ (23.6%), ‘Middle manager’ (11.5%), ‘Upper‐level management’ (8.9%), or ‘Chief Officer/Director’ (1.9%) were combined into the ‘manager’ category (45.9%).

### Results

We performed two repeated measures ANOVAs on ratings of masculinity and career commitment. The design was 2 (Target: self vs. junior women) within‐participant × 2 (Hierarchical Position: employee = −1 vs. manager = 1) between‐participants × perspective‐taking (continuous, grand‐mean centered). We carried out ANOVAs with repeated measures rather than linear regression with a difference score of self and junior ratings because in H1 we predict not only effects on self‐junior women comparisons but also an effect comparing self‐ratings of women managers and women employees on perceived masculinity. The correlations between the focal variables and exploratory variables are displayed in Table S1 of the Data S1.

#### Perceived masculinity

The main effect for target was significant, *F*(1, 310) = 63.63, *p* < .001, η_p_^2^ = 0.17, whereby women rated their own masculinity (*M* = 5.11, *SE* = 0.54) as higher than the masculinity of junior women (*M* = 4.47, *SE* = 0.06). Additionally, this main effect was qualified by a target × hierarchical position interaction, *F*(1, 310) = 19.97, *p* < .001, η_p_^2^ = 0.06. Simple slope analyses showed that, as predicted by Hypothesis 1a, managers rated themselves (*M* = 5.29, *SE* = 0.08) as higher in masculinity than they rated junior women (*M* = 4.29, *SE* = 0.09), *B* = 1.00, *SE* = 0.12, *p* < .001, 95% CI [0.77, 1.23], and that, although this self‐distancing effect also occurred for employees (self: *M* = 4.93, *SE* = 0.07, junior women: *M* = 4.65, *SE* = 0.08), *B* = 0.28, *SE* = 0.11, *p* = .010, 95% CI [0.07, 0.50], its effect size was almost only one tenth the magnitude (employees: η_p_^2^ = 0.02 vs. managers: η_p_^2^ = 0.19). Moreover, as predicted, managers rated themselves as more masculine (*M* = 5.29, *SE* = 0.08) than did employees (*M* = 4.93, *SE* = 0.07), *B* = 0.18, *SE* = 0.05, *p* < .001, 95% CI [0.07, 0.28]. In summary, in line with Hypothesis 1a, women managers showed greater distancing from junior women on the masculinity dimension than did women employees. Moreover, they considered themselves more masculine than women employees considered themselves.

**FIGURE 1:**
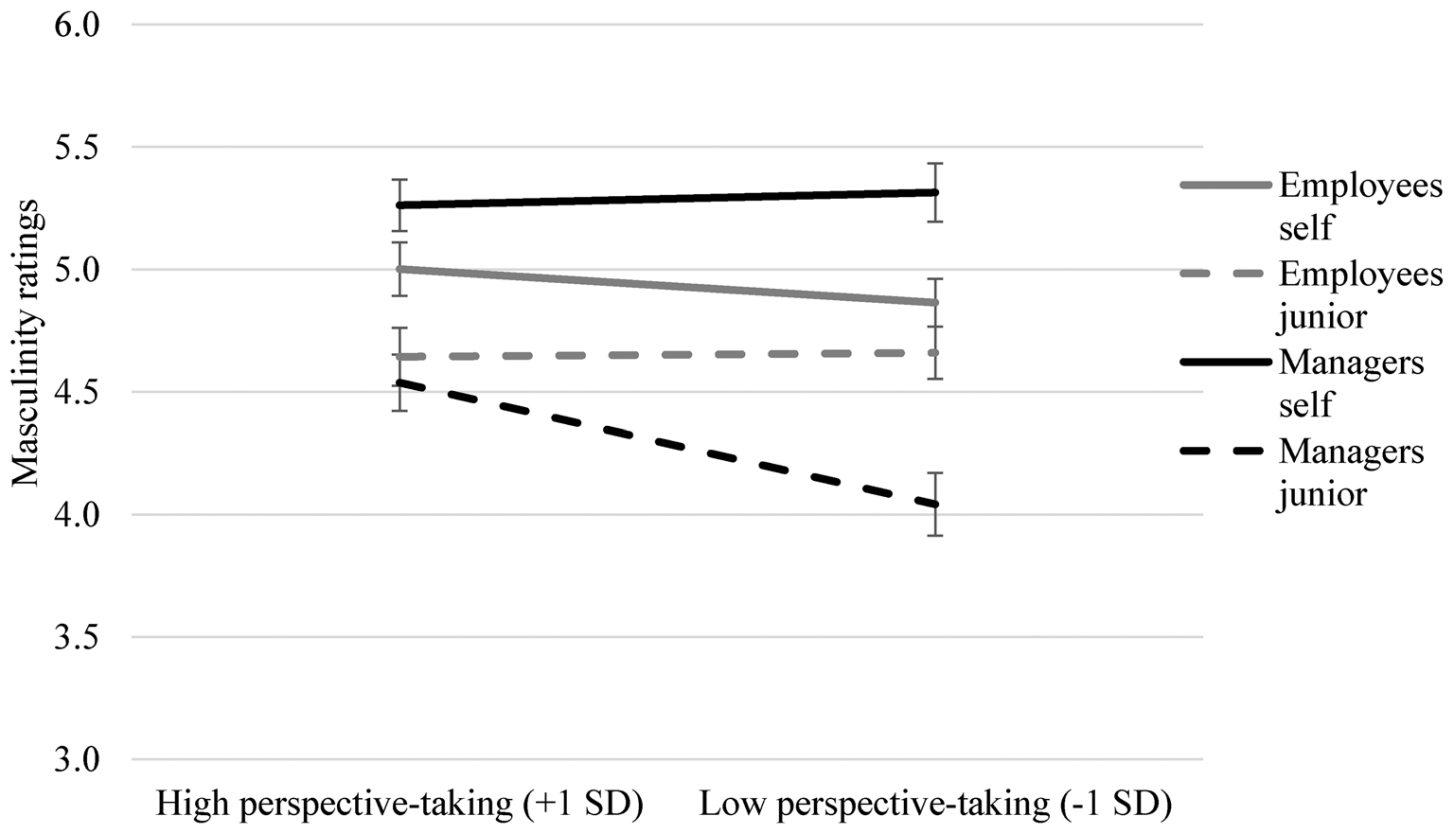
Masculinity Ratings of Self and Junior Women (7 Point Scale) by Employees and Managers With High Versus Low Perspective‐Taking Tendencies. Error Bars represent Standard Errors.

As predicted by Hypothesis 2a, the target × hierarchical position × perspective‐taking interaction was also significant, *F*(1, 310) = 4.76, *p* = .030, η_p_^2^ = 0.02. Decomposing this interaction within hierarchical position yielded a target × perspective‐taking interaction for managers, *F*(1, 310) = 5.39, *p* = .021, η_p_^2^ = 0.02, but not employees, *F*(1, 310) = 0.49, *p* = .483, η_p_^2^ < 0.01. Simple slopes showed that, among managers, high perspective‐taking (+1 SD) was associated with higher masculinity ratings of the self (*M* = 5.26, *SE* = 0.11) than of junior women (*M* = 4.54, *SE* = 0.12), *B* = 0.72, *SE* = 0.16, *p* < .001, 95% CI [0.42, 1.03]. However, this effect was more pronounced for low perspective‐taking (−1 SD, self: *M* = 5.31, *SE* = 0.12; junior: *M* = 4.04, *SE* = 0.13), *B* = 1.27, *SE* = 0.18, *p* < .001, 95% CI [0.92, 1.62], indicated by an effect size that was more than double the size for low perspective takers (η_p_^2^ = 0.14 vs. high perspective takers: η_p_^2^ = 0.07). Means are displayed in Figure 1.

#### Career commitment

The main effect of target was significant, *F*(1, 310) = 13.32, *p* < .001, η_p_^2^ = 0.04, whereby women reported lower career commitment for themselves (*M* = 4.34, *SE* = 0.09) than for junior women (*M* = 4.78, *SE* = 0.08). The target × hierarchical position interaction, *F*(1, 310) = 2.38, *p* = .124, η_p_^2^ < 0.01, predicted by Hypothesis 1b and the target × hierarchical position × perspective‐taking interaction, *F*(1, 310) = 1.63, *p* = .203, η_p_^2^ < 0.01, predicted by Hypothesis 2b, were not significant. The target × perspective‐taking interaction was also not significant (*p* = .079).

#### Support for affirmative action

We carried out an ANOVA with hierarchical position (manager = 1 vs. employee = −1) and perspective‐taking (continuous, grand‐mean centered) as independent variables and support for affirmative action as the dependent variable. The main effect of hierarchical position was significant, *F*(1, 310) = 5.26, *p* = .023, η_p_^2^ = 0.02, whereby as expected in Hypothesis 1c, employees indicated higher support for affirmative action (*M* = 5.92, *SE* = 0.07) than did managers (*M* = 5.67, *SE* = 0.08). The critical hierarchical position × perspective‐taking interaction predicted by Hypothesis 2c was not significant (*F*(1, 310) = 1.37, *p* = .243, η_p_^2^ < 0.01). However, the main effect of perspective‐taking was significant, *F*(1, 310) = 14.69, *p* < .001, η_p_^2^ = 0.05, showing that support for affirmative action was higher for high perspective‐takers (+1 SD, *M* = 6.00, *SE* = 0.08) than low perspective‐takers (−1 SD, *M* = 5.58, *SE* = 0.08) overall. Thus, perspective‐taking increased support for affirmative action for both managers and employees, but it did not decrease differences between the two groups.

### Discussion

The results from this correlational study are indicative of self‐distancing effects of women managers from junior women on ratings of masculinity, consistent with H1a, but, inconsistent with H1b, not career commitment. Women managers rated their own masculinity as higher than that of junior women, and women managers rated themselves as higher in masculinity than employee women rated themselves. Additionally, consistent with H2a, perspective‐taking among women managers attenuated self‐junior women distancing for masculinity ratings. For career commitment, inconsistent with H1b, both women managers and employees rated their own career commitment as lower than that of junior women. However, consistent with self‐group distancing responses and H1c, women managers indicated less support for affirmative action than did women employees. In contrast with our expectations as per H2c, perspective‐taking increased this support not only for women managers but also for employees.

## STUDY 2

We employed an experimental design to manipulate perspective‐taking and examine its causal effect on self‐group distancing responses of women managers towards junior women.

### Methods

#### Participants and design

The sample consisted of 347 women MTurk workers in the United States (mean age = 37.87, *SD* = 10.76) who completed an online survey and received 1 USD for compensation. From the original sample (*N* = 396), 35 participants were excluded from the analyses (3 participants did not agree for their responses to be used, 14 participants failed at least one of the attention checks, 18 participants indicated to be men although the survey explicitly invited women participants, 3 participants never had contact with a junior woman; some participants were excluded based on several of these criteria). Finally, 14 participants did not pass the comprehension checks and were excluded. Overall, 156 (45.0%) women were in an employee/non‐manager position, and 191 (55.0%) women indicated having a managerial position. The ethnic make‐up of the sample represents the U.S. population (12.4% African American, 5.2% Asian American, 24.5% White (Hispanic/Latino), 56.8% White (non‐Hispanic/Latino), 1.4% Native American, 1.2% had another ethnic background, and 0.9% preferred not to indicate their ethnicity). Participants worked on average 7.96 years in their organization (*SD* = 6.61) and on average 40.7 hours a week (*SD* = 6.55).

The study employed a 3 (perspective‐taking: junior women‐focus vs. self‐focus vs. other‐focus) × 2 (hierarchical position: manager vs. employee) between‐participants design. A priori power analysis, using G*power3 (Faul et al., 2007), indicated that to obtain 90% power for the expected medium effect size (*f* = 0.25) at *α* = .05, 204 participants were needed for a repeated ANOVA and 206 participants were needed for an ANOVA with 6 groups (df = 2). We recruited 400 participants to make sure to attain this number after exclusions (e.g., comprehension check, attention check).

#### Perspective‐taking manipulation

Participants were randomly assigned to one of three conditions. We manipulated perspective‐taking through a thought exercise (see Galinsky et al., 2014) that asked participants to focus on either junior women (junior women‐focus), themselves (self‐focus), or others (other‐focus); the latter two conditions serving as controls. In the *junior women‐focus* condition, participants were told to ‘Think about junior women. That is, try to reflect on how they are experiencing their careers and work‐life. What are the opportunities and/or challenges that they have encountered and may encounter in the future?’ In the *self‐focus* condition, they were told to ‘Think about yourself. That is, try to reflect on how you have been experiencing your career and work‐life. What are the opportunities and/or challenges that you have encountered and may encounter in the future?’ Finally, in the *other‐focus* condition, participants read ‘Think about ways to get to work. That is, try to reflect on how people get to work in the morning. What are the opportunities and/or challenges they have encountered and may encounter in the future on their way to work?’ We instructed participants to spend a couple of minutes writing down their thoughts. Comparing all three conditions allows us to pinpoint whether the specific act of taking the perspective of junior women (as opposed to any other perspective or self‐reflection) uniquely influences self‐distancing.

#### Measures

All participants were informed that ‘throughout this survey, we will use the term “junior” which refers to a person early in their career, no matter what age they are’. Measures were presented in the questionnaire in the same order as they appear below. Additional measures included for exploratory purposes and correlation Table S2 are reported in the Data S1.

##### Comprehension check

Following the thought exercise that exposed participants to one of the three perspective‐taking conditions, we asked participants to indicate the correct response out of two response options regarding their thoughts during the thought experiment. From the 361 participants retained after the other exclusions, *n* = 14 responded to at least one of these checks incorrectly and were thus excluded from the analyses. The first item gave two response options which were ‘I thought about the workplace’ (100% of the junior women‐focused and self‐focused conditions gave this correct answer) and ‘I thought about my way to work’ (91.5% in the other‐focused condition gave this correct answer). Then we asked participants to respond with ‘Yes’ versus ‘No’ to the following items ‘I objectively viewed this situation’ (88.2% in the other‐focused condition responded correctly ‘yes’); ‘I imagined my own feelings, thoughts, and experiences in this situation’ (98.5% in the self‐focused condition responded correctly ‘yes’) or ‘I imagined junior women's feelings, thoughts, and experiences in this situation’ (96.1% in the junior‐women‐focused condition responded correctly ‘yes’).

##### Self‐group distancing indicators

We used the same measures of perceived masculinity, perceived career commitment, and support for affirmative action as in Study 1.

##### Perceived masculinity

Participants indicated the degree to which masculinity was characteristic of themselves (*α* = .78, *M* = 4.63, *SD* = 1.28) and of junior women (*α* = .80, *M* = 4.42, *SD* = 1.18).

##### Perceived career commitment

Participants indicated the degree to which career commitment was characteristic of themselves (*α* = .92, *M* = 4.68, *SD* = 1.63) and of junior women (*α* = .91, *M* = 5.22, *SD* = 1.26).

##### Support for affirmative action

Participants indicated their support for affirmative action for junior women specifically (*α* = .92, *M* = 5.87, *SD* = 0.99).

##### Hierarchical position

Five categories assessed participants' hierarchical position: 45.0% employees, 19.0% were lower‐level managers, 27.7% middle managers, 6.6% upper‐level managers, and 1.7% executive/top managers. The latter four groups were combined into the group of managers.

### Results

We performed repeated‐measures ANOVAs with target ratings (self vs. junior women) for masculinity and for career commitment. We further carried out an ANOVA for the support of affirmative action variable. For all three analyses, we included the main effects of hierarchical position (employees = −1, managers =1) and perspective‐taking (coded as two orthogonal contrasts) and their interactions. Contrast 1 (C1) compared the perspective‐taking condition ‘junior women‐focus’ (coded 2) to the two control conditions, ‘self‐focus’ and ‘other‐focus’ (coded −1), used for testing predictions of H2. Contrast 2 (C2) opposed the ‘self‐focus’ (−1) to the ‘other‐focus’ (1) condition (‘junior women‐focus’ was coded 0).

#### Perceived masculinity

The main effect for target was significant, *F*(1, 341) = 5.96, *p* = .015, η_p_^2^ = 0.02, whereby women rated their own masculinity (*M* = 4.59, *SE* = 0.07) higher than the masculinity of junior women (*M* = 4.39, *SE* = 0.06). This main effect was further qualified by a target × hierarchical position interaction, *F*(1, 341) = 4.41, *p* = .037, η_p_^2^ = 0.01. Simple slope analyses showed that, as predicted by Hypothesis 1a, managers rated themselves (*M* = 5.01, *SE* = 0.09) as higher in masculinity than they rated junior women (*M* = 4.64, *SE* = 0.08), *B* = 0.37, *SE* = 0.11, *p* = .001, 95% CI [0.15, 0.58], whereas employees did not rate themselves differently from junior women (self: *M* = 4.17, *SE* = 0.10, junior: *M* = 4.15, *SE* = 0.09), *B* = 0.03, *SE* = 0.12, *p* = .818, 95% CI [−0.21, 0.26]. Moreover, as predicted, managers rated themselves as more masculine (*M* = 5.01, *SE* = 0.09) than did employees (*M* = 4.17, *SE* = 0.10), *B* = 0.40, *SE* = 0.06, *p* < .001, 95% CI [0.28, 0.53]. In summary, as per Hypothesis 1a, managers showed greater distancing from junior women on the masculinity dimension than did employees. Moreover, they considered themselves more masculine than employees considered themselves.

**FIGURE 2:**
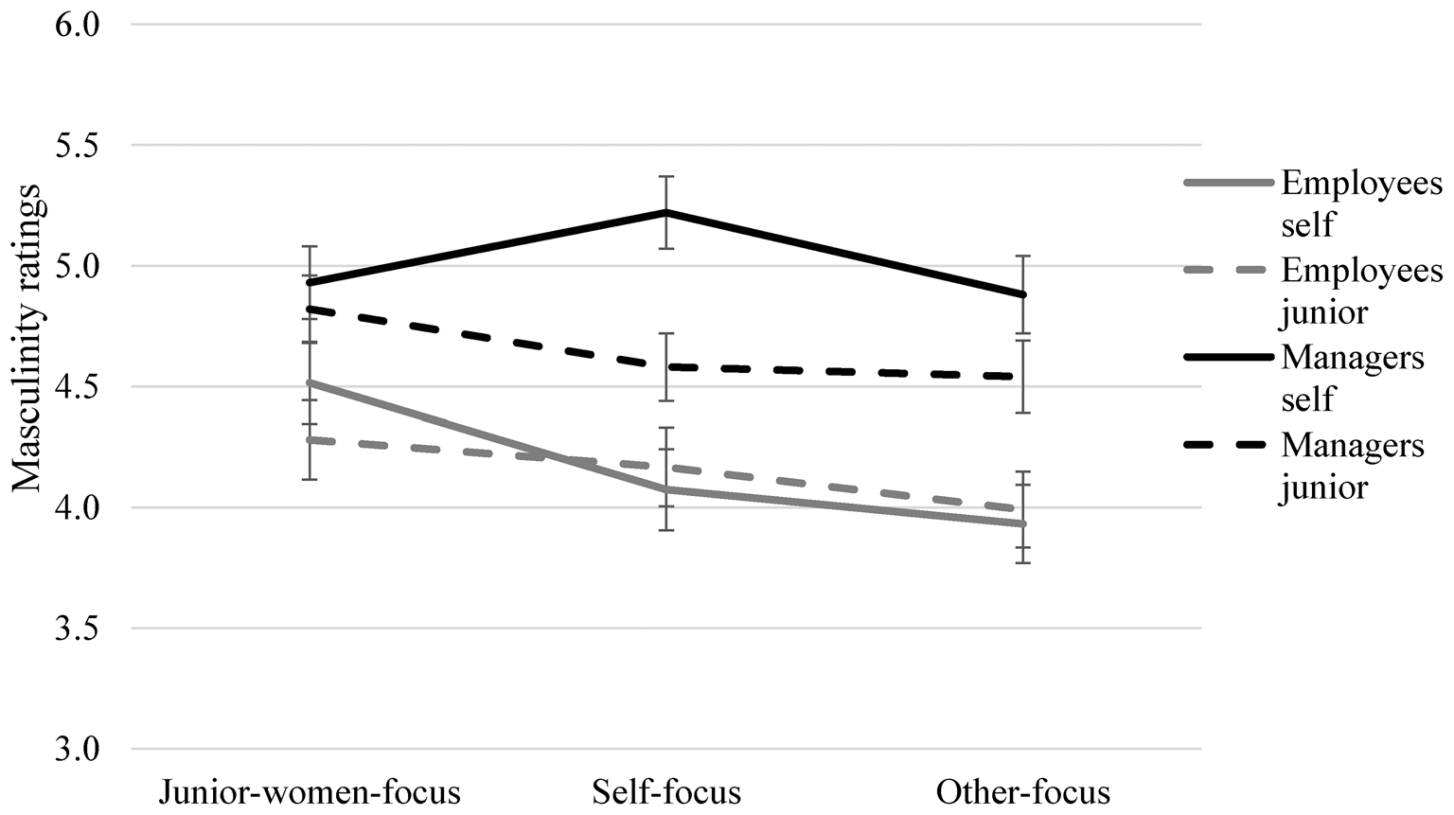
Masculinity Ratings of Self and Junior Women (7 Point Scale) for Employees and Managers Across Experimental Conditions. Error bars represent standard errors.

In addition, there was a target × hierarchical position × perspective‐taking C1 interaction, *F*(1, 341) = 4.02, *p* = .046, η_p_^2^ = 0.01. target × perspective‐taking C1 was *F*(1, 341) = 2.72, *p* = .100, η_p_^2^ = 0.008 for managers, and *F*(1, 341) = 1.48, *p* = .225, η_p_^2^ = 0.004 for employees. Simple slopes showed that, when managers adopted the perspective of junior women (junior women‐focus), their self‐ratings for masculinity did not differ from those for junior women (self: *M* = 4.93, *SE* = 0.15, junior: *M* = 4.82, *SE* = 0.14), *B* = 0.12, *SE* = 0.19, *p* = .533, 95% CI [−0.25, 0.48], whereas when managers did not (they either focused on themselves or others), they rated themselves as higher in masculinity than they rated junior women (self‐focus: self *M* = 5.22, *SE* = 0.15, junior *M* = 4.58, *SE* = 0.14; other‐focus: self *M* = 4.88, *SE* = 0.16, junior *M* = 4.54, *SE* = 0.15),^1^ *B* = 0.49, *SE* = 0.13, *p* = .001, 95% CI [0.23, 0.75]. None of these effects were significant for employees (*p*s > .266). Means are displayed in Figure 2. In summary, in line with Hypothesis 2a, taking the perspective of junior women attenuated the masculinity distancing effect found for managers in the control conditions (i.e., self‐focus combined with other‐focus).

#### Career commitment

The main effect of target was significant, *F*(1, 341) = 34.33, *p* < .001, η_p_^2^ = 0.09; women, regardless of their hierarchical position, ascribed lower levels of career commitment to themselves (*M* = 4.63, *SE* = 0.08) than to junior women (*M* = 5.20, *SE* = 0.07).

This main effect was qualified by a target × hierarchical position interaction, *F*(1, 341) = 9.71, *p* = .002, η_p_^2^ = 0.03. In contrast to Hypothesis 1b, managers ascribed lower levels of career commitment to themselves (*M* = 5.08, *SE* = 0.11) than to junior women (*M* = 5.35, *SE* = 0.09), *B* = −0.27, *SE* = 0.13, *p* = .042, 95% CI [−0.52, −0.01], which was also the case for employees (self: *M* = 4.18, *SE* = 0.13, junior: *M* = 5.05, *SE* = 0.10), *B* = −0.87, *SE* = 0.14, *p* < .001, 95% CI [−1.15, −0.59]. However, in line with H1b, managers ascribed higher levels of career commitment to themselves (*M* = 5.08, *SE* = 0.11) than did employees (*M* = 4.18, *SE* = 0.13), *B* = 0.45, *SE* = 0.08, *p* < .001, 95% CI [0.28, 0.61]. Thus, Hypothesis 1b was only partially supported.

The target × hierarchical position × perspective‐taking C1 interaction was not significant, *F*(1, 341) = 0.001, *p* = .997, η_p_^2^ < 0.01, thus Hypothesis 2b was not supported. None of the other interaction effects were significant, target × perspective‐taking C1: *F*(1, 341) = 0.35, *p* = .553, η_p_^2^ < 0.01; target × perspective‐taking C2: *F*(1, 341) = 0.288, *p* = .592, η_p_^2^ < 0.01; target × hierarchical position × perspective‐taking C2: *F*(1, 341) = 0.153, *p* = .695, η_p_^2^ < 0.01.

#### Support for affirmative action

The main effect for hierarchical position was not significant, *F*(1, 341) = 0.27, *p* = .606, η_p_^2^ < 0.01, thus, Hypothesis 1c was not supported. There was a main effect of perspective‐taking C1, *F*(1, 341) = 4.91, *p* = .027, η_p_^2^ = 0.01, whereby support was higher in the junior women‐focus condition (*M* = 6.05, *SE* = 0.09) than in the other two conditions (self‐focus: *M* = 5.93, *SE* = 0.09; other‐focus: *M* = 5.67, *SE* = 0.09). A main effect of perspective‐taking C2, *F*(1, 341) = 4.19, *p* = .041, η_p_^2^ = 0.01, showed that support was higher in the self‐focus condition than in the other‐focus condition. The hierarchical position × perspective‐taking C1 interaction was not significant, *F*(1, 341) = 2.53, *p* = .112, η_p_^2^ = 0.01 (nor was the perspective‐taking C2 × hierarchical position interaction, *F*(1, 341) = 0.07, *p* = .791, η_p_^2^ < 0.01). H2c was thus not supported.

### Discussion

Results from Study 2 are consistent with those of Study 1 and H1a in that self‐group distancing occurred for masculinity ratings, with managers ascribing more masculinity to themselves than to junior women (in their self‐ratings but also compared to employees' self‐perceptions). Of importance, consistent with Study 1 and H2a, perspective‐taking moderated the self‐group distancing response such that when managers adopted the perspective of junior women, masculinity self‐ratings and ratings of junior women did not differ, whereas self‐distancing from junior women was evident when managers either focused on themselves or others. In contrast to our expectations, for career commitment ratings, neither study found evidence of self‐group distancing in the expected direction nor a moderating effect of perspective‐taking. For affirmative action, we found that perspective‐taking increased support for affirmative action directed towards junior women, regardless of hierarchical level. Overall, we can conclude that the results of the two studies are comparable and that most importantly, adopting the view of junior women led to an attenuation of the self‐group distancing response regarding masculinity perceptions.

## GENERAL DISCUSSION

Consistent with the literature (Derks et al., 2016) and H1a, both studies found evidence of self‐group distancing responses for masculinity ratings such that women managers ascribed more masculinity to themselves than to junior women (in their self‐ratings but also compared to women employees’ self‐perceptions). In so doing, these women managers assimilate themselves into the higher‐status group of men and distance themselves from the group of junior women. Such an effect was not found for career commitment ratings (H1b). The expectation that affirmative action intentions should be lower for women managers than women employees (H1c), was only partially supported, as the effect only occurred in Study 1 but not in Study 2. Novel for the literature on how to combat the self‐group distancing phenomenon, both studies found that perspective‐taking reduced these self‐group distancing responses for masculinity ratings, thereby supporting H2a. No attenuation occurred for career commitment (H2b), and perspective‐taking related positively to affirmative action intentions for both women managers and employees, contrary to expectations (H2c).

These findings emerged across different research methods– a correlational design in Study 1, which measured women's perspective‐taking, and an experimental design in Study 2, which manipulated perspective‐taking. In Study 1, women managers with higher levels of perspective‐taking showed less self‐junior women distancing for masculinity ratings than women managers with lower levels of perspective‐taking. In Study 2, women managers adopting the perspective of junior women, rated themselves no different than they rated junior women, whereas self‐distancing from junior women was evident when women managers either focused on themselves or others. Overall, both studies’ findings suggest that perspective‐taking is a fruitful strategy for (i) reducing self‐group distancingresponses for perceived masculinity and (ii) increasing support for affirmative action support for both women managers and employees. Moreover, Study 2 showed that, focusing on either junior women, or the self, led to increased intentions to support affirmative action measures. This suggests that focusing on women's barriers in the workplace encountered either by women themselves or imagined for junior women led to increased support of affirmative actions.

It is noteworthy that although the current research found that women distanced themselves from junior women by self‐ascribing masculine traits, in both studies, counter to expectations, women reported lower career commitment for themselves than for junior women. Prior work shows that self‐group distancing in perceptions of career commitment (whereby women rate their commitment higher for themselves than junior women) was only found for women who did not highly identify with other women and perceived gender discrimination (Derks et al., 2016). As such, in the current studies, women managers may still feel attached to their gender ingroup, even if they self‐describe as also having the traits of their second identity, that of a manager. Future research is needed to better understand how and when women respond with self‐distancing on different dimensions. Additionally, self‐distancing responses may vary across measurement dimensions (attitudes, identification, behaviour, or intentions; see Chipeaux et al., 2024).

For support of affirmative action, it was interesting to see that women managers supported them less compared to women employees in Study 1 which focused on “women” but not in Study 2 which focused on ‘junior women’. Junior women may be seen as more deserving of affirmative action (as they are also seen as higher on career commitment), thus reducing women managers’ opposition to such policies in Study 2. Additionally, state (Study 1) and experimentally induced perspective‐taking (Study 2) or focus on the self (Study 2) increased women's support for such measures, regardless of their hierarchical position. The stronger affirmative action in the self‐focus condition may have prompted some women to reflect on the gender discrimination they face at work, potentially driving them to mobilize for gender equality.

### Theoretical and practical contributions

Taken together, the present research has important theoretical and practical implications. First, our results provide theoretical insights regarding two contradictory views about women's approaches to helping junior women in the workplace (Maume, 2011). One view that stresses solidarity and sisterhood between women (see Mavin, 2006) sees women managers supporting junior women's careers and thus acting as change agents, whereas another view that highlights the social identity threat experienced by women in male‐dominated organizations (e.g., Kulich et al., 2015; Paustian‐Underdahl et al., 2017) points to women who have achieved high‐status, high‐power roles showing ingroup distancing instead.

Our findings suggest that the degree to which women managers engage in self‐distancing from junior women–when assessed as masculinity perceptions–depends on whether they take the perspective of these junior women or not. According to the literature, those in power tend to be more egocentrically preoccupied and less aware of the social consequences of their behaviour (Galinsky et al., 2006; Keltner et al., 2003; Lammers et al., 2010), and power combined with perspective‐taking might assist the powerful in using their agency for good and thus yield beneficial effects (Galinsky et al., 2014). Our research advances efforts to understand how perspective‐taking by those in power positions can yield synergistic effects, particularly for women managers. Women managers are often working in organizational settings that are male‐dominated and thus potentially pose a threat to their own social identity. Such situations of identity threat enhance women managers' tendency for self‐distancing from their former ingroup of other, more junior women. Notably, in our study, women managers who engaged in perspective‐taking showed less distancing by rating junior women as higher in masculinity and more similar to themselves. Thus, “power with perspective‐taking allows people to reach their destination without crashing into others along the way” (p. 633, Galinsky et al., 2014).

Second, our findings contribute to recent work that identifies guidelines for interventions to combat the self‐group distancing phenomenon, which can help improve support mechanisms for junior women. Given that self‐group distancing is likely to emerge as an individual mobility response when the social identity of women in male‐dominated organizations is threatened, the ideal scenario would involve removing the threat altogether by having companies in which more women hold leadership positions and/or in which diversity‐friendly climates exist (Paustian‐Underdahl et al., 2017). Overall, our findings thus present yet another alternative to combat self‐group distancing responses which is for women managers to engage in perspective‐taking–that is to put themselves into the shoes of junior women when thinking about challenges and opportunities in relation to careers and work life. Our findings show that both a state of high perspective‐taking as well as an intervention where women are being asked to actively adopt the perspective of junior women reduce self‐distancing from junior women among women managers for masculinity. This knowledge can be leveraged for perspective‐taking training interventions in organizations not only to combat self‐group distancing responses but also to improve interpersonal outcomes (e.g., Calvard et al., 2021; Todd et al., 2011), whilst being mindful that under certain circumstances perspective‐taking might also increase stereotyping (e.g., Skorinko & Sinclair, 2013).

### Limitations

A potential limitation is that our study did not include participants' responses to both junior women and junior men; however, previous evidence has already shown that women managers are more biased against junior women than junior men (e.g., Faniko et al., 2021). Finally, future research could further explore the link between interpersonal contact and perspective‐taking (Schilke & Huang, 2018) as higher levels of interpersonal contact of managers with their employees, for example, due to a smaller span of control (number of employees who report to a supervisor), might foster perspective‐taking and thus reduce self‐group distancing responses among women managers.

## CONCLUSION

Our paper started with the quote that ‘Empowered Women Empower Women’, which expresses the hope that women who have achieved social mobility and power in organizations will empower, support, and act as inspirational role models to other, more junior women. Yet, this paper highlighted two conflicting views in the current literature according to which women might show supportive behaviours versus self‐distancing from junior women. The findings from our two studies illuminated self‐distancing responses among women managers for ratings of masculinity; however, they also provide an optimistic view whereby perspective‐taking can attenuate these self‐group distancing responses.

## AUTHOR CONTRIBUTIONS

**Janine Bosak:** Conceptualization; methodology; data curation; formal analysis; project administration; writing – original draft. **Clara Kulich:** Conceptualization; methodology; data curation; formal analysis; visualization; project administration; writing – original draft; writing – review and editing. **Samantha C. Paustian‐Underdahl:** Conceptualization; methodology; data curation; writing – review and editing. **Rachelle Borg Dingli:** Conceptualization; methodology; data curation.

## CONFLICT OF INTEREST STATEMENT

The manuscript is not being simultaneously submitted elsewhere. The authors are not aware of any conflict of interest in relation to the paper. Prior to commencing the studies, ethical approval was obtained from Dublin City University Business School for Study 1 and the Faculty of Psychology and Educational Sciences at the University of Geneva for Study 2 (PSE.20190604.10). Finally, the manuscript has been read and approved by all the named authors.

## Supporting information


Data S1.

## Data Availability

Our Data Transparency statement in the paper as per p. 10 reads as follows: All data, analysis code, and research materials are available by emailing the corresponding author for Study 1 and on OSF for Study 2 (https://osf.io/vc9ed). Study 2's design, hypotheses and analysis were pre‐registered (https://osf.io/ajknu) and additional pre‐registered exploratory questions were formulated for the measures included in the Supplementary Materials. Ethical approval was obtained for Study 1 and for Study 2.

## Footnotes

^1^
Comparisons per condition: junior‐focus *F*(1, 341) = 0.39, *p* = .533, η_p_^2^ = 0.00; self‐focus *F*(1, 341) = 12.16, *p* = .001, η_p_^2^ = 0.03, and other‐focus *F*(1, 341) = 3.15, *p* = .077, η_p_^2^ = 0.01.

## References

[bjso12812-bib-0001] Aronson, J. , Cohen, G. , & Nail, P. R. (1999). Self‐affirmation theory: An update and appraisal. In E. Harmon‐Jones & J. Mills (Eds.), Cognitive dissonance: Progress on a pivotal theory in social psychology (pp. 127–147). US: American Psychological Association. 10.1037/10318–006

[bjso12812-bib-0003] Bem, S. L. (1974). The measurement of psychological androgyny. Journal of Consulting and Clinical Psychology, 42, 155–162. 10.1037/h0036215 4823550

[bjso12812-bib-0004] Branscombe, N. R. , Ellemers, N. , Spears, R. , & Doosje, B. (1999). The context and content of social identity threat. In N. Ellemers , R. Spears , & B. Doosje (Eds.), Social identity: Context, commitment, content (Vol. 77, pp. 35–58). Blackwell. 10.1037//0022–3514.77.1.135

[bjso12812-bib-0005] Braun, S. , Stegmann, S. , Hernandez Bark, A. S. , Junker, N. M. , & van Dick, R. (2017). Think manager—Think male, think follower—Think female: Gender bias in implicit followership theories. Journal of Applied Social Psychology, 47(7), 377–388. 10.1111/jasp.12445

[bjso12812-bib-0006] Brion, S. , & Anderson, C. (2013). The loss of power: How illusions of alliance contribute to powerholders' downfall. Organizational Behavior and Human Decision Processes, 121, 129–139. 10.1016/j.obhdp.2013.01.005

[bjso12812-bib-0007] Calvard, T. , Cherlin, E. , Brewster, A. , & Curry, L. (2021). Building perspective‐taking as an organizational capability: A change intervention in a health care setting. Journal of Management Inquiry, 32, 35–49. 10.1177/10564926211039014

[bjso12812-bib-0008] Chipeaux, M. , Kulich, C. , Iacoviello, V. , Politi, E. , & Lorenzi‐Cioldi, F. (2024). Anticipated and achieved individual mobility amongst Portuguese immigrants in Switzerland: Social identity adjustment and inter‐minority relations. Social Psychological Bulletin, 19, 1–25. 10.32872/spb.9465

[bjso12812-bib-0009] Chipeaux, M. , Kulich, C. , Vincenzo, I. , & Lorenzi‐Cioldi, F. (2017). I want, therefore I am‐anticipated upward mobility reduces ingroup concern. Frontiers in Psychology, 8, 1451. 10.3389/fpsyg.2017.01451 28894431 PMC5581401

[bjso12812-bib-0010] Cialdini, R. B. , Brown, S. L. , Lewis, B. P. , Luce, C. , & Neuberg, S. L. (1997). Reinterpreting the empathy‐altruism relationship: When one into one equals oneness. Journal of Personality and Social Psychology, 73, 481–494. 10.1037/0022-3514.73.3.481 9294898

[bjso12812-bib-0011] Clore, G. L. , & Jeffery, K. M. (1972). Emotional role playing, attitude change, and attraction toward a disabled person. Journal of Personality and Social Psychology, 23(1), 105–111. 10.1037/h0032867 4261340

[bjso12812-bib-0012] Davis, M. H. (1980). A multidimensional approach to individual differences in empathy. JSAS Catalog of Selected Documents in Psychology, 10, 85.

[bjso12812-bib-0013] Davis, M. H. (1983). The effects of dispositional empathy on emotional reactions and helping: A multidimensional approach. Journal of Personality, 51, 167–184. 10.1111/j.1467-6494.1983.tb00860.x

[bjso12812-bib-0014] Davis, M. H. , Conklin, L. , Smith, A. , & Luce, C. (1996). Effect of perspective taking on the cognitive representation of persons: A merging of self and other. Journal of Personality and Social Psychology, 70, 713–726. 10.1037/0022-3514.70.4.713 8636894

[bjso12812-bib-0015] Derks, B. , Scheepers, D. , Van Laar, C. , & Ellemers, N. (2011). The threat vs. challenge of car parking for women: How self‐ and group affirmation affect cardiovascular responses. Journal of Experimental Social Psychology, 47, 178–183. 10.1016/j.jesp.2010.08.016

[bjso12812-bib-0016] Derks, B. , van Laar, C. , & Ellemers, N. (2009). Working for the self or working for the group: How self‐versus group affirmation affects collective behavior in low‐status groups. Journal of Personality and Social Psychology, 96, 183–202. 10.1037/a0013068 19210074

[bjso12812-bib-0017] Derks, B. , van Laar, C. , & Ellemers, N. (2016). The queen bee phenomenon: Why women leaders distance themselves from junior women. The Leadership Quarterly, 27, 456–469. 10.1016/j.leaqua.2015.12.007

[bjso12812-bib-0019] Devine, P. G. , Forscher, P. S. , Austin, A. J. , & Cox, W. T. (2012). Long‐term reduction in implicit race bias: A prejudice habit‐breaking intervention. Journal of Experimental Social Psychology, 48, 1267–1278. 10.1016/j.jesp.2012.06.003 23524616 PMC3603687

[bjso12812-bib-0020] Eagly, A. H. , & Karau, S. J. (2002). Role congruity theory of prejudice toward female leaders. Psychological Review, 109, 573–598. 10.1037/0033-295X.109.3.573 12088246

[bjso12812-bib-0021] Ellemers, N. (2001). Individual upward mobility and the perceived legitimacy of intergroup relations. In J. T. Jost & B. Major (Eds.), The psychology of legitimacy: Emerging perspectives on ideology, justice, and intergroup relations (pp. 205–222). Cambridge University Press.

[bjso12812-bib-0022] Ellemers, N. , de Gilder, D. , & Van den Heuvel, H. (1998). Career‐oriented versus team‐oriented commitment and behavior at work. Journal of Applied Psychology, 83(5), 717–730. 10.1037/0021-9010.83.5.717

[bjso12812-bib-0023] Ellemers, N. , Rink, F. , Derks, B. , & Ryan, M. K. (2012). Women in high places: When and why promoting women into toppositions can harm them individually or as a group (and how to prevent this). Research In Organizational Behavior, 32, 163–187. 10.1016/j.riob.2012.10.003

[bjso12812-bib-0024] Ely, R. J. (1995). The power in demography: Women's social constructions of gender identity at work. The Academy of Management Journal, 38, 589–634. 10.2307/256740

[bjso12812-bib-0025] Faniko, K. , Ellemers, N. , & Derks, B. (2016). The Queen Bee phenomenon in Academia 15 years after: Does it still exist, and if so, why? British Journal of Social Psychology, 46, 903–913. 10.1002/ejsp.2198 PMC8246980 32696985

[bjso12812-bib-0054] Faniko, K. , Ellemers, N. , & Derks, B. (2021). The Queen Bee phenomenon in Academia 15 years after: Does it still exist, and if so, why? British Journal of Social Psychology, 60(2), 383–399. 10.1111/bjso.12408 32696985 PMC8246980

[bjso12812-bib-0026] Faniko, K. , Ellemers, N. , Derks, B. , & Lorenzi‐Cioldi, F. (2017). Nothing changes, really: Why women who break through the glass ceiling end up reinforcing it. Personality and Social Psychology Bulletin, 43, 638–651. 10.1177/0146167217695551 28903635 PMC5414903

[bjso12812-bib-0027] Faul, F. , Erdfelder, E. , Lang, A. G. , Lang, A. G. , & Buchner, A. (2007). G*power 3: A flexible statistical power analysis program for the social, behavioral, and biomedical sciences. Behavior Research Methods, 39, 175–191. 10.3758/BF03193146 17695343

[bjso12812-bib-0028] Fiske, S. T. (1993). Controlling other people: The impact of power on stereotyping. American Psychologist, 48, 621–628. 10.1037/0003-066X.48.6.621 8328729

[bjso12812-bib-0029] Galinsky, A. D. , Magee, J. C. , Inesi, M. E. , & Gruenfeld, D. H. (2006). Power and perspective not taken. Psychological Science, 17, 1068–1074. 10.1111/j.1467-9280.2006.01824.x 17201789

[bjso12812-bib-0030] Galinsky, A. D. , Magee, J. C. , Rus, D. , Rothman, N. B. , & Todd, A. R. (2014). Acceleration with steering: The synergistic benefits of combining power and perspective‐taking. Social Psychological and Personality Science, 5, 627–635. 10.1177/1948550613519685

[bjso12812-bib-0031] Galinsky, A. D. , & Moskowitz, G. B. (2000). Perspective‐taking: Decreasing stereotype expression, stereotype accessibility, and in‐group favoritism. Journal of Personality and Social Psychology, 78, 708–724. 10.1037//0022-3514.78.4.708 10794375

[bjso12812-bib-0032] Keltner, D. , Gruenfeld, D. H. , & Anderson, C. (2003). Power, approach, and inhibition. Psychological Review, 110, 265–284. 10.1037/0033-295X.110.2.265 12747524

[bjso12812-bib-0033] Kohlberg, L. (1976). Moral stages and moralization: The cognitive developmental approach. In T. Lickona (Ed.), Moral development and behavior (pp. 31–53). Holt, Rinehart & Winston.

[bjso12812-bib-0034] Kraus, M. W. , Côté, S. , & Keltner, D. (2010). Social class, contextualism, and empathic accuracy. Psychological Science, 21, 1716–1723. 10.1177/0956797610387613 20974714

[bjso12812-bib-0035] Kulich, C. , Lorenzi‐Cioldi, F. , & Iacoviello, V. (2015). Moving across status lines: Low concern for the ingroup and group identification. Journal of Social Issues, 71, 453–475. 10.1111/josi.12123

[bjso12812-bib-0036] Lammers, J. , Stapel, D. A. , & Galinsky, A. D. (2010). Power increases hypocrisy: Moralizing in reasoning, immorality in behavior. Psychological Science, 21(5), 737–744. 10.1177/0956797610368810 20483854

[bjso12812-bib-0037] Lee, F. , & Tiedens, L. Z. (2001). Who's being served? “self‐serving” attributions in social hierarchies. Organizational Behavior and Human Decision Processes, 84, 254–287. 10.1006/obhd.2000.2925 11277672

[bjso12812-bib-0038] Li, Z. , & Edwards, J. A. (2021). The relationship between system justification and perspective‐taking and empathy. Personality and Social Psychology Bulletin, 47, 106–117. 10.1177/0146167220921041 32400294

[bjso12812-bib-0039] Magee, J. C. , & Smith, P. K. (2013). The social distance theory of power. Personality and Social Psychology Review, 17, 158–186. 10.1177/1088868312472732 23348983

[bjso12812-bib-0040] Maume, D. J. (2011). Meet the new boss…same as the old boss? Female supervisors and subordinate career prospects. Social Science Research, 40, 287–298. 10.1016/j.ssresearch.2010.05.001 PMC3003918 21180397

[bjso12812-bib-0041] Mavin, S. (2006). Venus envy: Problematizing solidarity behaviour and queen bees. Women in Management Review, 21, 264–276. 10.1108/09649420610666579

[bjso12812-bib-0042] Paustian‐Underdahl, S. C. , King, E. B. , Rogelberg, S. G. , Kulich, C. , & Gentry, W. A. (2017). Perceptions of supervisor support: Resolving paradoxical patterns across gender and race. Journal of Occupational and Organizational Psychology, 90, 436–457. 10.1111/joop.12179

[bjso12812-bib-0043] Ragins, B. R. , & Scandura, T. A. (1994). Gender differences in expected outcomes of mentoring relationships. Academy of Management Journal, 37, 957–971. 10.2307/256606

[bjso12812-bib-0044] Schilke, O. , & Huang, L. (2018). Worthy of swift trust? How brief interpersonal contact affects trust accuracy. Journal of Applied Psychology, 103(11), 1181–1197. 10.1037/apl0000321 29963894

[bjso12812-bib-0045] Sheppard, L. D. , & Aquino, K. (2013). Much ado about nothing? Observers' problematization of women's same‐sex conflict at work. The Academy of Management Perspectives, 27(1), 52–62. 10.5465/amp.2012.0005

[bjso12812-bib-0046] Skorinko, J. L. , & Sinclair, S. A. (2013). Perspective taking can increase stereotyping: The role of apparent stereotype confirmation. Journal of Experimental Social Psychology, 49, 10–18. 10.1016/j.jesp.2012.07.009

[bjso12812-bib-0047] Staines, G. L. , Tavris, C. , & Jayaratne, T. E. (1974). The Queen bee syndrome. Psychology Today, 7, 55–60. 10.1037/E400562009-003

[bjso12812-bib-0055] Tajfel, H . & Turner, J.C .(1986). The Social Identity Theory of Intergroup Behavior. In: S. Worchel & W. G. Austin (Eds.), Psychology of Intergroup Relation (pp. 7– 24). Chicago: Hall Publishers.

[bjso12812-bib-0049] Todd, A. R. , Bodenhausen, G. V. , Richeson, J. A. , & Galinsky, A. D. (2011). Perspective taking combats automatic expressions of racial bias. Journal of Personality and Social Psychology, 100, 1027–1042. 10.1037/a0022308 21381852

[bjso12812-bib-0050] Tougas, F. , & Veilleux, F. (1988). The influence of identification, collective relative deprivation, and procedure of implementation on women's response to affirmative action: A causal modeling approach. Canadian Journal of Behavioural Science/Revue Canadienne Des Sciences du Comportement, 20(1), 15–28. 10.1037/h0079920

[bjso12812-bib-0051] van Kleef, G. A. , Oveis, C. , van der Löwe, I. , LuoKogan, A. , Goetz, J. , & Keltner, D. (2008). Power, distress, and compassion: Turning a blind eye to the suffering of others. Psychological Science, 19, 1315–1322. 10.1111/j.1467-9280.2008.02241.x 19121143

[bjso12812-bib-0052] Vial, A. C. , & Napier, J. L. (2017). High power mindsets reduce gender identification and benevolent sexism among women (but not men). Journal of Experimental Social Psychology, 68, 162–170. 10.1016/j.jesp.2016.06.012

[bjso12812-bib-0053] Wang, C. S. , Tai, K. , Ku, G. , & Galinsky, A. D. (2014). Perspective‐taking increases willingness to engage in intergroup contact. PLoS One, 9(1), e85681. 10.1371/journal.pone.0085681 24465648 PMC3899073

