# Supplementary material for: The role of perspective‐taking in attenuating self‐group distancing in women managers
Source: Br J Soc Psychol. 2024 Oct 28;64(2):e12812. doi: 10.1111/bjso.12812 (PMC11927376; doi:10.1111/bjso.12812)
Supplement: Supplementary file 1 — Data S1. [file BJSO-64-0-s001.docx]

# Supplementary Materials

**Study 1 Focal Measures and Items**

**Perceived Masculinity.** Four attributes (assertive, having leadership abilities, willing to take risks, dominant) taken from Bem’s Sex Role Inventory (Bem, 1974, see also Faniko et al., 2016) rated for self (α = .71, *M* = 5.09, *SD* = 0.96), and junior women (α = .76, *M* = 4.50, *SD* = 1.09).

**Perceived Career Commitment.** Three items from Ellemers et al. (1998), see also Faniko et al. (2016); including “My career is one of the most important things in my life”; “My career plays a central role in my life” and “The ambitions in my life mainly have to do with my career” - rated for self (α = .90, *M* = 4.36, *SD* = 1.62, e.g., “My career is one of the most important things in my life”) and junior women (α = .96, *M* = 4.78, *SD* = 1.45, e.g., “The career is one of the most important things in their life”).

**Support for Affirmative Action.** Seven items from Tougas and Veilleux (1988; α = .90, *M* = 5.79, *SD* = 0.97): “Given equal qualifications, I agree that the hiring of women in sectors where they are underrepresented should be facilitated”; “Given equal qualifications, I support that the promotion of women in sectors where they are underrepresented should be facilitated”; I am in favor of implementing training programs designed to give women access to non-traditional jobs”; “I am in favor of facilitating women's integration in formal and informal power networks”; “I am in favor of informing as well as making management and personnel sensitive to women's disadvantageous situation in the work force”; “I am in favor of informing women of career possibilities in the organization” and “I am in favor of implementing an equality of access program”.

### Perspective-Taking

Seven items from the Interpersonal Reactivity Index (IRI, Davis, 1980; see for example, Wang et al., 2014; α = .73, *M* = 5.29, *SD* = 0.74); “I sometimes find it difficult to see things from the “other person’s” point of view”, “I try to look at everybody’s side of a disagreement before I make a decision”; “I sometimes try to understand my friends better by imaging how things look from their perspective”; “If I’m sure I’m right about something, I don’t waste much time listening to other people’s arguments”; “I believe that here are two sides to every question and try to look at them both”; “When I’m upset at someone, I usually try to “put myself in his shoes” for a while”; and “Before criticizing somebody, I try to imagine how I would feel if I were in their place.”

**Study 1 Exploratory Measures and Items**

**Leadership Aspiration.** Single item presented amongst demographic questions.: **“**I aspire to have a position with leadership responsibilities in the future.” (*M* = 4.91, *SD* = 1.62)

**Gender Climate of Gender Equality.** Four items from Paustian-Underdahl et al. (2017): “At work others make jokes or negative commentaries about women”; “At work, I get the impression that being female may be a limitation”; “At work, I feel that others exclude women from some activities”; and “At work, I get the sense that there are limited opportunities for advancement for female employees” (α = .88, *M* = 2.62, *SD* = 1.43).

**Competitive Psychological Climate**. Four items from Fletcher et al. (2008): “Everybody is concerned with being the top performer.”; “My manager frequently compares my performance with that of my coworkers.”; “The amount of recognition you get in this company depends on how you perform compared to others”; and “My coworkers frequently compare their performance with mine” (α = .77, *M* = 2.98, *SD* = 1.28)

**Concern for Women.** Two items from Kulich et al. (2015) and Hersby et al. (2009): “I feel concerned by the problems experienced by women during their career”, “Women exaggerate the difficulties they encounter in the workplace” (*r* = .34, *M* = 4.85, *SD* = 1.22)

**Perceived Collective Discrimination.** Two items from Kulich et al. (2015): “Women are discriminated against in their professional career, compared to men”, “It is easier for a man to have a successful career than it is for a woman” (*r* = .66, *M* = 4.60, *SD* = 1.56)

**Perceived Meritocracy.** Two items from Kulich et al. (2015): “In the workplace, promotions reflect competencies”, “In the workplace, those who make the greatest effort are promoted” (*r* = .74, *M* = 3.62, *SD* = 1.59)

**Empathic Concern.** Seven items from the interpersonal reactivity index (Davis, 1980): “I often have tender, concerned feelings for people less fortunate than me”; “Sometimes I don’t feel very sorry for other people when they are having problems”; “When I see someone being taken advantage of, I feel kind of protective forwards them”; “Other people’s misfortunes do not usually disturb me a great deal”; “When I see someone being treated unfairly, I sometimes don’t feel very much pity for them.”; “I am often quite touched by things that I see happen”; and “I would describe myself as a pretty soft-hearted person.”

(α = .76, *M* = 5.57, *SD* = 0.79)

**Table 1**

*Correlations Between Key Concepts and Exploratory Measures (Study 1, N = 314)*

|  |  | 1 | 2 | 3 | 4 | 5 | 6 | 7 | 8 | 9 | 10 | 11 | 12 | 13 | 14 |
| --- | --- | --- | --- | --- | --- | --- | --- | --- | --- | --- | --- | --- | --- | --- | --- |
| 1 | Hierarchical Status (dichotomous) |  |  |  |  |  |  |  |  |  |  |  |  |  |  |
| 2 | Perspective Taking | .11 |  |  |  |  |  |  |  |  |  |  |  |  |  |
| 3 | Age (years) | .21^**^ | .04 |  |  |  |  |  |  |  |  |  |  |  |  |
| 4 | Masculinity: Self | .19^**^ | .05 | -.09 |  |  |  |  |  |  |  |  |  |  |  |
| 5 | Masculinity: Junior women | -.16^**^ | .09 | -.11^*^ | -.04 |  |  |  |  |  |  |  |  |  |  |
| 6 | Career commitment: Self | .09 | .18^**^ | -.12^*^ | .38^**^ | -.04 |  |  |  |  |  |  |  |  |  |
| 7 | Career commitment: Junior women | -.03 | .05 | -.10 | .16^**^ | .24^**^ | .25^**^ |  |  |  |  |  |  |  |  |
| 8 | Support for affirmative action | -.10 | .20^**^ | .05 | .10 | .03 | .16^**^ | .09 |  |  |  |  |  |  |  |
| 9 | Leadership Aspirations | .12^*^ | .04 | -.35^**^ | .43^**^ | .00 | .43^**^ | .14^*^ | .11^*^ |  |  |  |  |  |  |
| 10 | Perceived Climate of Gender Inequity | .03 | -.11^*^ | -.06 | .08 | -.04 | .11 | -.03 | .03 | .08 |  |  |  |  |  |
| 11 | Competitive Psychological Climate | -.02 | -.04 | -.02 | .06 | .04 | .10 | -.06 | .00 | .01 | .44^**^ |  |  |  |  |
| 12 | Concern for Women | -.07 | .07 | .01 | .05 | .10 | .20^**^ | .16^**^ | .32^**^ | .14^*^ | .24^**^ | .07 |  |  |  |
| 13 | Perceived Collective Discrimination | .03 | -.03 | -.13^*^ | .08 | .00 | .03 | .05 | .14^*^ | .20^**^ | .45^**^ | .17^**^ | .42^**^ |  |  |
| 14 | Perceived Meritocracy | -.07 | -.06 | -.11^*^ | .06 | .12^*^ | .02 | .05 | .05 | .09 | -.19^**^ | -.04 | -.02 | -.16^**^ |  |
| 15 | Empathic Concern | .09 | .46^**^ | .06 | .10 | .06 | .15^**^ | .10 | .21^**^ | .04 | -.17^**^ | -.10 | .14^*^ | .00 | -.06 |

*Note.* * < .05, ** <.01

**Study 1 Focal Measures and Items**

**Self-group Distancing Indicators.** The same measures to rate perceived masculinity for themselves(α = .78, *M* = 4.63, *SD* = 1.28) and junior women (α = .80, *M* = 4.42, *SD* = 1.18), perceived career commitment for themselves (α = .92, *M* = 4.68, *SD* = 1.63) and for junior women (α = .91, *M* = 5.22, *SD* = 1.26), and support for affirmative action (α = .92, *M* = 5.87, *SD* = 0.99) as in Study 1 (for the full list of items see “Study 1 Focal Measures and Items” in the supplementary materials). In contrast to study 1 however the items used in study 2 made specific reference to ‘junior women’ rather than just ‘women’.

**Study 2 Additional Analyses**

**Sensitivity.** Sensitivity analyses using G*power 3 (Faul et al., 2007) indicated that with the final sample size of Study 2 (*N* = 347), α = .05, and 90% desired power, the minimum effect size that we could detect is f = 0.19 (or η_p_^2^ = 0.035) for the repeated ANOVAs and the ANOVA.

**Masculinity.** The full decomposition of the Target × Hierarchical Position × Perspective-taking C1 interaction reported for masculinity ratings shows in addition to the effects reported in the paper the Perspective-taking C1 × Hierarchical position was *B* = -0.11, *SE* = 0.05, *p* = .023, 95% CI [-0.20, -0.01] for the self, and *B* = 0.01, *SE* = 0.05, *p* = .830, 95% CI [-0.08, 0.10] for junior women. This shows that the differences between managers and employees’ self-ratings are smaller in the junior-focus condition than in the other two, which is in the spirit of our hypotheses. Target × Hierarchical position was *F*(1, 341) = 0.04, *p* = .851, η_p_^2^ < 0.001 for Perspective taking C1, and *F*(1, 341) = 8.31, *p* = .004, η_p_^2^ = 0.024 for Perspective taking C2.

**Study 2 Exploratory Measures and Items**

**Femininity.** Four traits (tolerant, warm, understanding, sensitive to the needs of others) from Faniko et al. (2017) applied to the self (α = .80, *M* = 5.75, *SD* = 0.88) and to junior women (α = .86, *M* = 5.27, *SD* = 1.09).

**Expected Performance.** Three performance items: “Junior women perform well in my organization/ in the workplace.”, “Junior women fit in on the job in my organization/ in the workplace”, and “Junior women in my organization/ in the workplace are promotable”, (α = .87, *M* = 5.49, *SD* = 1.06)

**Concern for the Ingroup**. Two items from Kulich et al. (2015) and Hersby et al. (2009): "I feel concerned by the problems experienced by junior women during their career in my organization/team/the workplace”, "Junior women exaggerate the difficulties they encounter in my organization/team/the workplace”, (*r* = -.20, *M* = 4.81, *SD* = 1.42)

**Support Towards Junior Women**. Five items from Ryan et al. (2012); “I would show junior women the ‘ins and outs’ of the organization”; “I would like to be a mentor to junior women”; I would help junior women succeed in the organization”; “I would like to work with junior women”; and “I would introduce junior women to people I know in management” (α = .89, *M* = 5.87, *SD* = 1.01).

**Identification with Junior Women**. Three items "I feel strong ties with junior women”, "I think that junior women have a lot to be proud of”, “I identify with junior women” (α = .85, *M* = 5.47, *SD* = 1.22)

**Perspective Taking.** Seven items from Interpersonal Reactivity Index (IRI, Davis, 1980; see for example, Wang et al., 2014) used in Study 1 (α = .86, *M* = 5.41, *SD* = 1.02); for the full list of items see “Study 1 Focal Measures and Items” in the supplementary materials

**Professional Aspirations.** Three items from the psychological (“My aspirations are very high in terms of professional recognition and achievement”; “For me, the problems associated with holding a position of leadership outweigh the benefits.”; and “I would like to access a position in which I can have a greater influence on political and/or strategic decisions.”) and three items from the behavioral dimension (“I have sought feedback on my professional performance.”; “I have tried to plan my career path.”; and “ I have improved my skills in order to be more competitive for promotion.”) of a scale from Tharenou and Terry (1998), (α = .71, *M* = 5.11, *SD* = 0.97)

**Self-sacrifice.** Scale with six items from Faniko et al. (2017): “I have devoted my time to work instead of being with my family or friends”; “I have cancelled, shortened, or delayed personal vacations to be able to meet work commitments”; “Because of my career, it happened that I attached less importance to my values and convictions”; “I have accepted offensive remarks in order not to jeopardize my career; “It happened that I prioritize my career over my personal relationships”; and “I adapted my decision whether or not, and when, to have children to the requirements of my career” (α = .89, *M* = 3.66, *SD* = 1.63)

**Gender Diversity Climate**. Five items from Virick and Geer (2012); “My organization ensures that junior women are not overlooked in promotion decisions”; “My organization provides adequate networking opportunities for junior women”; “My organization provides adequate training opportunities for junior women”; “The organization provides clearly defined career opportunities/career paths for junior women”; “My organization provides opportunities for visible assignments and cross-functional experiences for junior women.” (α = .93, *M* = 4.92, *SD* = 1.40)

**Perceived Meritocracy.** Two items from Kulich et al. (2015), (*r* = .79, *M* = 5.12, *SD* = 1.55); “Overall, promotions in my organization/team reflect competencies.”; and “Overall, those who make the greatest efforts are promoted in my organization/team.”

**Competitive Psychological Climate**. Four items from Fletcher et al. (2008), (α = .85, *M* = 4.33, *SD* = 1.51): “My manager frequently compares my performance with that of my coworkers.”; “The amount of recognition you get in this company depends on how you perform compared to others.”; “Everybody is concerned with being the top performer.” and “My coworkers frequently compare their performance with mine.”

**Bibliography**

Bem, S. L. (1974). The measurement of psychological androgyny. *Journal of Consulting and Clinical Psychology, 42*, 155–162. [https://doi.org/10.1037/h0036215](https://psycnet.apa.org/doi/10.1037/h0036215)

Davis, M. H. (1980). A multidimensional approach to individual differences in empathy. *JSAS Catalog of Selected Documents in Psychology, 10,* 85.

Ellemers, N., de Gilder, D. & Van den Heuvel, H. (1998). Career-oriented versus team-oriented commitment and behavior at work. *Journal of Applied Psychology*, 83(5), 717. <https://doi-org.dcu.idm.oclc.org/10.1037/0021-9010.83.5.717>

Faniko, K., Ellemers, N., & Derks, B. (2016). Queen Bees and Alpha Males: Are successful women more competitive than successful men? *European Journal of Social Psychology, 46*, 903–913. [https://doi.org/10.1002/ejsp.2198](https://psycnet.apa.org/doi/10.1002/ejsp.2198)

Faniko, K., Ellemers, N., Derks, B., & Lorenzi-Cioldi, F. (2017). Nothing changes, really: Why women who break through the glass ceiling end up reinforcing it. *Personality and Social Psychology Bulletin*, *43*, 638–651. <https://doi.org/10.1177%2F0146167217695551>

Faul, F., Erdfelder, E., Lang, AG. *et al.* G*Power 3: A flexible statistical power analysis program for the social, behavioral, and biomedical sciences. *Behavior Research Methods* 39, 175–191 (2007). <https://doi.org/10.3758/BF03193146>

Fletcher, T. D., Major, D. A., & Davis, D. D. (2008). The interactive relationship of competitive climate and trait competitiveness with workplace attitudes, stress, and performance. *Journal of Organizational Behavior*, *29*(7), 899-922. <https://doi.org/10.1002/job.503>

Hersby, M. D., Ryan, M. K., & Jetten, J. (2009). Getting together to get ahead: The impact of social structure on women's networking. *British Journal of Management*, *20*(4), 415-430. <https://doi.org/10.1111/j.1467-8551.2008.00604.x>

Kulich, C., Lorenzi-Cioldi, F., & Iacoviello, V. (2015). Moving across status lines: Low concern for the ingroup and group identification. *Journal of Social Issues*, *71*, 453–475. https://psycnet.apa.org/doi/10.1111/josi.12123

Paustian‐Underdahl, S. C., King, E. B., Rogelberg, S. G., Kulich, C., & Gentry, W. A. (2017). Perceptions of supervisor support: Resolving paradoxical patterns across gender and race. *Journal of Occupational and Organizational Psychology, 90*, 436–457. [https://doi.org/10.1111/joop.12179](https://psycnet.apa.org/doi/10.1111/joop.12179)

Ryan, K. M., King, E. B., Adis, C., V. Gulick, L. M., Peddie, C., & Hargraves, R. (2012). Exploring the asymmetrical effects of gender tokenism on supervisor-subordinate relationships. *Journal of Applied Social Psychology*, *42*, E56-E102. https://doi.org/10.1111/j.1559-1816.2012.01025.x

Tharenou, P., & Terry, D. J. (1998). Reliability and validity of scores on scales to measure managerial aspirations. *Educational and Psychological Measurement, 58*(3), 475–492. [https://doi.org/10.1177/0013164498058003008](https://psycnet.apa.org/doi/10.1177/0013164498058003008)

Tougas, F., & Veilleux, F. (1988). The influence of identification, collective relative deprivation, and procedure of implementation on women's response to affirmative action: A causal modeling approach. *Canadian Journal of Behavioural Science / Revue canadienne des sciences du comportement*, 20(1), 15–28. <https://doi.org/10.1037/h0079920>

Virick, M. & Greer, C.R. (2012) Gender diversity in leadership succession: Preparing for the future. *Human Resource Management*, *51*, 575-600.
<https://doi.org/10.1002/hrm.21487>

Wang, C. S., Tai, K. Ku, G. & Galinsky, A. D. (2014). Perspective-taking increases willingness to engage in intergroup contact. *PLoS One*, Jan 22;9(1):e85681. https://doi.org/10.1371/journal.pone.0085681

**Table 2**

*Correlations Between Key Concepts and Exploratory Measures (Study 2, N = 347)*

|  |  | 1 | 2 | 3 | 4 | 5 | 6 | 7 | 8 | 9 | 10 | 11 | 12 | 13 | 14 | 15 | 16 | 17 | 18 |
| --- | --- | --- | --- | --- | --- | --- | --- | --- | --- | --- | --- | --- | --- | --- | --- | --- | --- | --- | --- |
| 1 | Hierarchical Position |  |  |  |  |  |  |  |  |  |  |  |  |  |  |  |  |  |  |
| 2 | Age | .01 |  |  |  |  |  |  |  |  |  |  |  |  |  |  |  |  |  |
| 3 | Masculinity: Self | .33** | <.001 |  |  |  |  |  |  |  |  |  |  |  |  |  |  |  |  |
| 4 | Masculinity: Junior women | .21** | -.06 | .26** |  |  |  |  |  |  |  |  |  |  |  |  |  |  |  |
| 5 | Career commitment: Self | .28** | -.12* | .36** | .24** |  |  |  |  |  |  |  |  |  |  |  |  |  |  |
| 6 | Career commitment: Junior women | .12* | -.01 | .04 | .38** | .24** |  |  |  |  |  |  |  |  |  |  |  |  |  |
| 7 | Affirmative Action | -.02 | .01 | .15** | .18** | .15** | .23** |  |  |  |  |  |  |  |  |  |  |  |  |
| 8 | Femininity: Self | .09 | .05 | .20** | .22** | .18** | .16** | .33** |  |  |  |  |  |  |  |  |  |  |  |
| 9 | Femininity: Junior women | .12* | -.09 | .27** | .13* | .24** | .21** | .33** | .38** |  |  |  |  |  |  |  |  |  |  |
| 10 | Expected Performance | .07 | -.05 | .01 | .39** | .28** | .47** | .38** | .21** | .31** |  |  |  |  |  |  |  |  |  |
| 11 | Concern for the ingroup | .01 | -.07 | -.05 | .10 | .09 | .17** | .54** | .13* | .24** | .26** |  |  |  |  |  |  |  |  |
| 12 | Support Towards Junior Women | .12* | .02 | .29** | .19** | .25** | .18** | .58** | .42** | .36** | .31** | .39** |  |  |  |  |  |  |  |
| 13 | Identification with Junior Women | .08 | -.15** | .15** | .31** | .31** | .37** | .52** | .28** | .41** | .45** | .49** | .62** |  |  |  |  |  |  |
| 14 | Perspective Taking | -.03 | .04 | .05 | .05 | .02 | .02 | .37** | .54** | .15** | .19** | .10 | .40** | .17** |  |  |  |  |  |
| 15 | Professional Aspirations | .31** | -.07 | .43** | .22** | .50** | .16** | .37** | .31** | .33** | .26** | .32** | .52** | .46** | .30** |  |  |  |  |
| 16 | Self-sacrifice | .16** | -.26** | .23** | .19** | .50** | .16** | -.04 | .01 | .14** | .06 | .07 | .03 | .25** | -.13* | .26** |  |  |  |
| 17 | Gender Diversity Climate | .13* | .01 | .18** | .30** | .21** | .19** | .04 | .17** | .11* | .37** | -.14** | .16** | .17** | .09 | .19** | .04 |  |  |
| 18 | Perceived Meritocracy | .11 | .04 | .10 | .21** | .20** | .21** | .03 | .14** | .10 | .31** | -.16** | .16** | .18** | .07 | .21** | .04 | .78** |  |
| 19 | Competitive Psychological Climate | .16** | -.13* | .21** | .28** | .26** | .21** | -.03 | .20** | .09 | .10 | -.03 | .16** | .28** | .00 | .25** | .40** | .25** | .29** |

*Note.* * < .05, ** <.01
